# Current Methodological Pitfalls and Caveats in the Assessment of Exercise-Induced Changes in Peripheral Brain-Derived Neurotrophic Factor: How Result Reproducibility Can Be Improved

**DOI:** 10.3389/fnrgo.2021.678541

**Published:** 2021-05-28

**Authors:** Chiara Nicolini, Aimee J. Nelson

**Affiliations:** Department of Kinesiology, McMaster University, Hamilton, ON, Canada

**Keywords:** exercise, BDNF, neuroplasticity, mobility, motor cortex, serum, plasma, ELISA

## Abstract

Neural mechanisms, such as enhanced neuroplasticity within the motor system, underpin exercise-induced motor improvements. Being a key mediator of motor plasticity, brain-derived neurotrophic factor (BDNF) is likely to play an important role in mediating exercise positive effects on motor function. Difficulties in assessing brain BDNF levels in humans have drawn attention to quantification of blood BDNF and raise the question of whether peripheral BDNF contributes to exercise-related motor improvements. Methodological and non-methodological factors influence measurements of blood BDNF introducing a substantial variability that complicates result interpretation and leads to inconsistencies among studies. Here, we discuss methodology-related issues and approaches emerging from current findings to reduce variability and increase result reproducibility.

## Introduction

**Figure 1:**
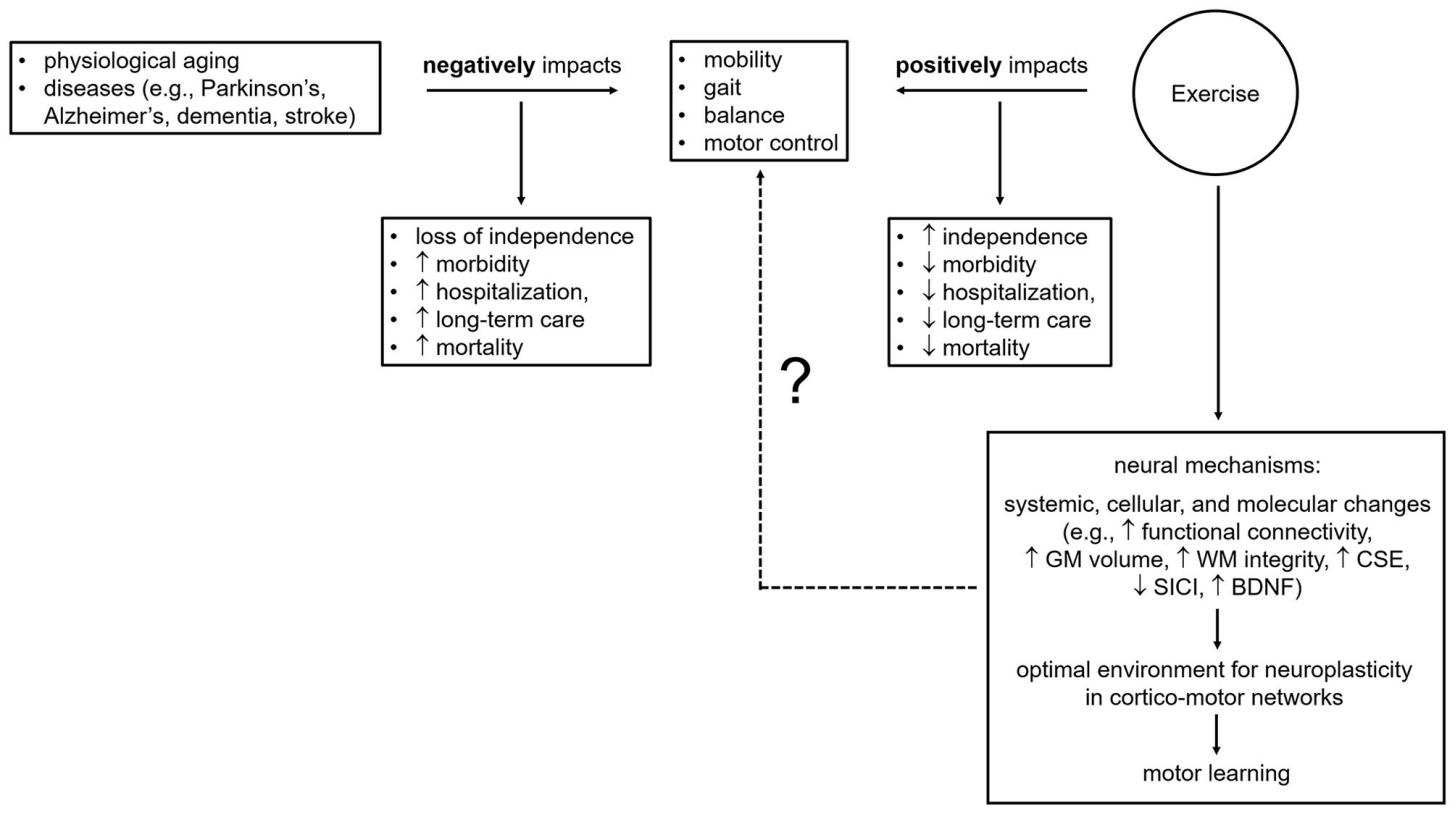
Effects of age, illness, and exercise on motor outcomes and brain mechanisms underpinning exercise-related motor benefits. BDNF, brain-derived neurotrophic factor; CSE, corticospinal excitability; GM, gray matter; SICI, short-interval intracortical inhibition; WM, white matter; ?, knowledge gaps to be addressed and filled by future research.

A growing body of evidence shows that exercise helps attenuate disease-related motor impairments (Stein, 2004; Marigold et al., 2005; Crizzle and Newhouse, 2006; Herman et al., 2007; Gobbi et al., 2009; Quaney et al., 2009; Ridgel et al., 2009; Cooke et al., 2010; Hauer et al., 2012; Vreugdenhil et al., 2012; Gomes de Melo Coelho et al., 2013b; Pitkälä et al., 2013; van der Kolk and King, 2013; Schwenk et al., 2014a,b; Duchesne et al., 2015; Dennett et al., 2016; Cugusi et al., 2019; Gretebeck et al., 2019; Linder et al., 2019) and maintain motor function in aging adults (Rikli and Edwards, 1991; Buckwalter, 1997; Campbell et al., 1999; Visser et al., 2002; Means et al., 2005; Pahor et al., 2014; Bolandzadeh et al., 2015; Brach et al., 2017; Hsu et al., 2017a,b; Hübner et al., 2018). Age- and disease-associated motor impairments include reduced balance, motor control, gait speed and stride length, altered rhythm, rigidity, and slow movements and lead to a decline in physical functioning and mobility, and consequently to falls and fall-related injuries, which result in a loss of independence, morbidity, and mortality (Overstall et al., 1977; Winter et al., 1990; Tinetti and Williams, 1998; Sterling et al., 2001; Jørgensen et al., 2002; Grimbergen et al., 2004; Todd and Skelton, 2004; Weerdesteyn et al., 2008; Ioannidis et al., 2009; Blankevoort et al., 2010; Deandrea et al., 2010; Tinetti and Kumar, 2010; Fasano et al., 2017; Lach et al., 2017; Xu et al., 2018; Osoba et al., 2019; Zhang et al., 2019). Since exercise improves mobility, gait speed and rhythmicity, stride length, postural reflexes, balance, and motor control in the elderly, stroke survivors, and individuals with Parkinson's, Alzheimer's, or dementia (Marigold et al., 2005; Crizzle and Newhouse, 2006; Herman et al., 2007; Goodwin et al., 2008; Gobbi et al., 2009; Quaney et al., 2009; Blankevoort et al., 2010; Brienesse and Emerson, 2013; van der Kolk and King, 2013; Schwenk et al., 2014b; Mehrholz et al., 2015), it appears effective at prolonging the ability to perform daily activities and at reducing injuries, morbidity, and mortality related to falls. However, our understanding of *how* exercise improves mobility, balance, motor control, and gait parameters such as speed, rhythmicity, and stride length needs to be expanded (Figure 1). It is now widely acknowledged that exercise benefits mobility not just by improving physiological function, such as muscle strength and balance (Robertson et al., 2002; Liu-Ambrose et al., 2008, 2013), but also through neural mechanisms (e.g., enhanced neuroplasticity, maintenance of white and gray matter integrity and volume in motor brain areas) (Shepherd, 2001; Colcombe et al., 2003, 2006; Forrester et al., 2008; Quaney et al., 2009; Petzinger et al., 2010, 2013; Mang et al., 2013; Perrey, 2013; Duchesne et al., 2016; Hirsch et al., 2016; Nepveu et al., 2017; Steib et al., 2018). Nevertheless, studies providing direct evidence are few (Fisher et al., 2008; Skriver et al., 2014; Bolandzadeh et al., 2015; Ostadan et al., 2016; Hsu et al., 2017a,b; Dal Maso et al., 2018; Hübner et al., 2018; Lehmann et al., 2020). Specifically, Hsu et al. (2017a,b) showed that exercise-induced maintenance of functional connectivity within fronto-parietal networks, which are involved in motor planning and execution, was linked to improved mobility in elderly subjects with mild subcortical ischemic vascular cognitive impairment. Bolandzadeh et al. (2015) found that exercise-related reduction in the progression of white matter lesions was associated with gait speed maintenance in older women. Further, Fisher et al. (2008) and Hübner et al. (2018) reported that exercise-induced improvements in gait parameters (i.e., speed, step and stride length, hip and ankle joint excursion) and fine motor control (as measured by a precision grip force modulation task) were accompanied with primary motor cortex excitability changes (i.e., longer cortical silent period) in Parkinson's patients and enhanced frontal and sensorimotor cortex activity (i.e., decreases in EEG task-related power in the beta band, 13–30 Hz) in healthy, elderly subjects, respectively. Last, greater motor skill acquisition and retention as well as learning of a new motor task have been shown to be associated with larger changes in neural activity, corticospinal excitability, GABA_A_-mediated inhibition (i.e., short-interval intracortical inhibition, SICI), white matter microstructure, and brain-derived neurotrophic factor (BNDF) levels in young, healthy adults following exercise (Skriver et al., 2014; Ostadan et al., 2016; Stavrinos and Coxon, 2017; Dal Maso et al., 2018; Lehmann et al., 2020). Of note, the ability of exercise to promote motor skill learning and retention has important implications during rehabilitation, for example post-stroke, for the recovery of motor disabilities which hinder independent living (Mang et al., 2013). Promisingly, Quaney et al. (2009) reported that exercise lead to improvements in motor learning which in turn, translated into improved fine motor control (i.e., greater predictive force modulation to grasp and lift an object) in chronic stroke survivors. Despite these promising findings supporting the notion that neural substrates (e.g., enhanced neuroplasticity within motor networks promoting motor learning) mediate exercise positive effects on mobility, gait, balance, and motor control, the mechanistic links between exercise and motor improvements largely remains to be unraveled (Figure 1). Indeed, as exercise-induced structural and functional brain changes as well as motor outcomes have been mainly assessed in separate studies, it is pivotal for shedding light onto the neural correlates of exercise-induced motor improvements that these different levels of analysis (i.e., systemic, cellular, molecular, and behavioral) are carried out within the same study in future research.

**Figure 2:**
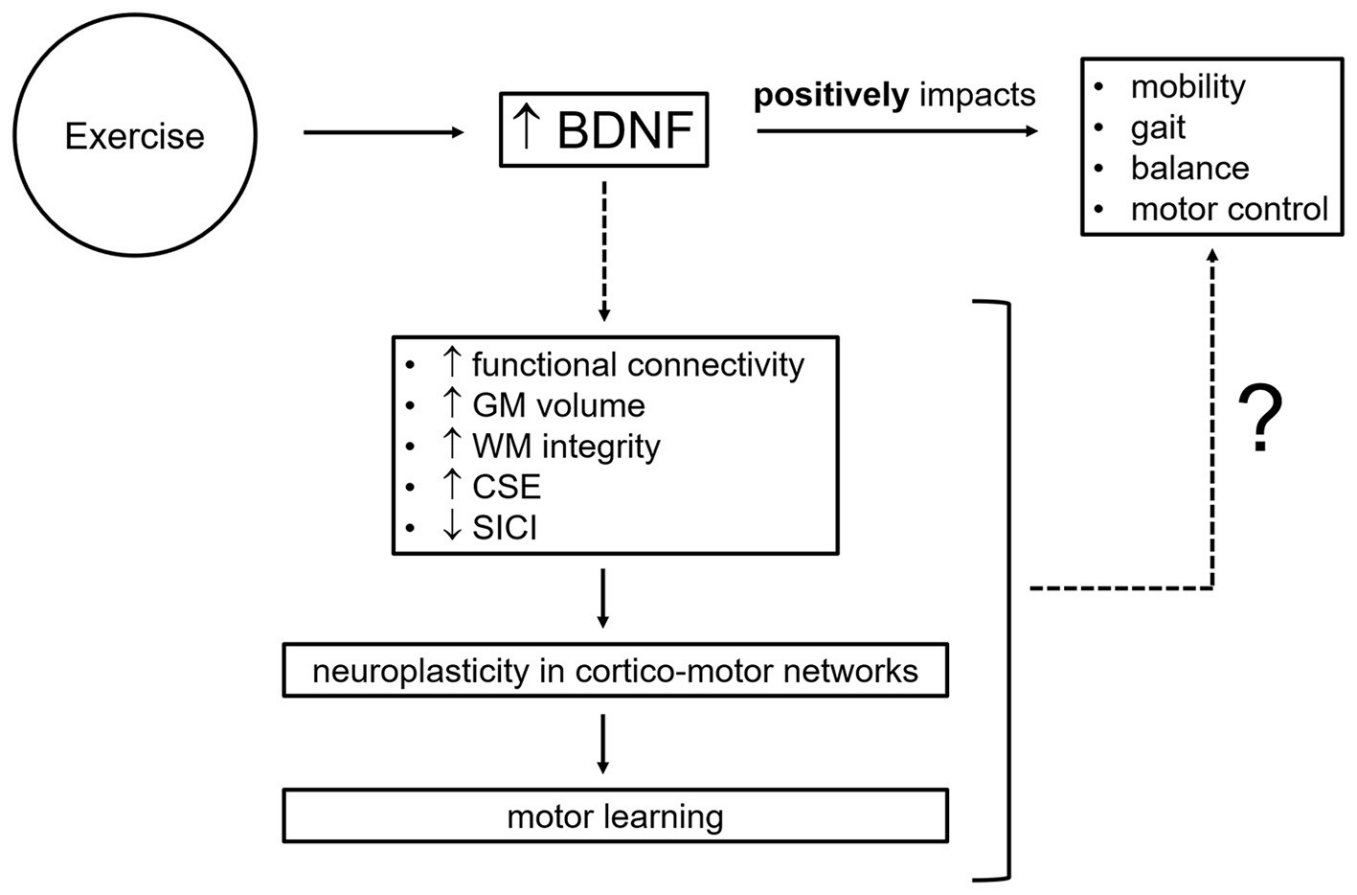
Potential neural mechanisms by which brain-derived neurotrophic factor mediates exercise-related motor improvements. BDNF, brain-derived neurotrophic factor; CSE, corticospinal excitability; GM, gray matter; SICI, short-interval intracortical inhibition; WM, white matter; ?, knowledge gaps to be addressed and filled by future research.

Approaches used to garner evidence of exercise-linked systemic, cellular, and molecular changes include quantification of molecular markers (e.g., BDNF, osteocalcin, and irisin) in blood using enzyme-linked immunosorbent assays (ELISAs), assessment of corticospinal excitability using single-pulse transcranial magnetic stimulation (TMS), evaluation of GABAergic [i.e., short-interval intracortical inhibition, SICI, and long-interval intracortical inhibition, LICI, reflecting GABA_A_-mediated inhibition and GABA_B_-mediated inhibition, respectively (Rossini et al., 2015; Ziemann et al., 2015)] and glutamatergic (i.e., intracortical facilitation, ICF, and short-interval intracortical facilitation, SICF) motor circuits via paired-pulse TMS paradigms, and measurement of white matter (WM) tract integrity and neurometabolite concentrations [e.g., inhibitory neurotransmitter γ-aminobutyric acid (GABA) and excitatory neurotransmitter glutamate] with magnetic resonance techniques such as diffusion tensor imaging (DTI) and magnetic resonance spectroscopy (MRS). Here, we focus on brain-derived neurotrophic factor, likely a key mediator of the positive effects of exercise on mobility, gait, balance, and motor control by promoting neuroplasticity within motor brain circuits which, in turn, facilitates motor learning (Figure 2). We discuss limitations and future avenues for the investigation of BDNF contribution to exercise-related motor outcomes. Understanding the mechanisms through which aerobic exercise promotes brain plasticity and ultimately leads to motor benefits is critical for the design of exercise protocols effective in the prevention, delay, attenuation, and recovery of age- and disease-related motor impairments.

## Brain-Derived Neurotrophic Factor

The molecular mediators of exercise-induced brain changes, such as enhanced neuroplasticity, which by facilitating motor learning likely contributes to exercise-related motor improvements (i.e., mobility, gait, balance, and motor control), are still largely unknown. Molecular markers that have been identified as likely candidates include the neurotrophin brain-derived neurotrophic factor (BDNF), the growth factor insulin-like growth factor 1 (IGF-1), the bone-derived hormone osteocalcin (OCN), and lastly myokines cathepsin B and irisin. In this review, special attention is paid to BDNF, a member of the neurotrophin family which includes nerve growth factor (NGF), neurotrophin 3, and neurotrophin 4/5 (Barde, 1994; Lewin and Barde, 1996; Hallböök, 1999). Since its discovery in 1982 (Barde et al., 1982), BDNF has been demonstrated to be essential for normal brain development and adult brain function with alterations in its levels accompanying neurological and psychiatric disorders (McAllister et al., 1995; Huang and Reichardt, 2001; McAllister, 2002; Tyler et al., 2002; Binder and Scharfman, 2004; Bramham and Messaoudi, 2005; Kuipers and Bramham, 2006; Brunoni et al., 2008; Chapleau et al., 2009; Fahnestock, 2011; Fernandes et al., 2011; Green et al., 2011; Carlino et al., 2013; Park and Poo, 2013; Molendijk et al., 2014; Fahnestock and Nicolini, 2015; Hempstead, 2015; Armeanu et al., 2017; Illarioshkin et al., 2018; Mohammadi et al., 2018; Numakawa et al., 2018; Di Carlo et al., 2019; Huang et al., 2019; Lima Giacobbo et al., 2019; Ng et al., 2019). BDNF is synthesized as a 32-kDa precursor, called proBDNF, which is subsequently cleaved, either intracellularly (e.g., by serine protease furin and prohormone convertases) or extracellularly (e.g., by matrix metalloproteases or serine protease plasmin), into a 14-kDa mature form (Seidah et al., 1996; Lee et al., 2001; Mowla et al., 2001; Pang et al., 2004, 2016). ProBDNF is not an inactive precursor but has distinct and opposite functions from mature BDNF (Lu et al., 2005). Specifically, proBDNF reduces neuronal survival, neurite growth, and dendritic spine formation and induces neuronal apoptosis and long-term depression via p75 neurotrophin receptor (p75NTR) (Teng et al., 2005; Woo et al., 2005; Koshimizu et al., 2009). Mature BDNF promotes neuronal differentiation and survival, neurite growth, neural circuit formation, function, and maintenance, synaptogenesis, and synaptic plasticity, both during development and throughout adulthood, via tyrosine kinase receptor tropomyosin-related kinase B (TrkB) (McAllister et al., 1999; Schinder and Poo, 2000; Huang and Reichardt, 2001; Poo, 2001; McAllister, 2002; Binder and Scharfman, 2004; Waterhouse and Xu, 2009; Park and Poo, 2013; Leal et al., 2014, 2017; Lu et al., 2014; Gibon and Barker, 2017; Kowiański et al., 2018). Through a different cleavage site, proBDNF can be converted into a 28-kDa protein (truncated BDNF) (Seidah et al., 1999; Mowla et al., 2001), which is not an intermediate product in the proteolytic processing of proBDNF into mature BDNF, but whose biological activities are still unknown. Of note, an altered balance of the three BDNF proteolytic isoforms (i.e., pro, truncated, mature) has been observed in patients with schizophrenia and idiopathic autism (Carlino et al., 2011; Garcia et al., 2012), suggesting that all three isoforms play important roles for normal brain function.

### Brain-Derived Neurotrophic Factor and Exercise

A number of studies have shown that exercise elevates peripheral BDNF concentrations (Gold et al., 2003; Ferris et al., 2007; Goekint et al., 2008; Tang et al., 2008; Zoladz et al., 2008; Rasmussen et al., 2009; Knaepen et al., 2010; Seifert et al., 2010; Yarrow et al., 2010; Zoladz and Pilc, 2010; Bos et al., 2011; Griffin et al., 2011; Rojas Vega et al., 2011; Cho et al., 2012; Heyman et al., 2012; Schmidt-Kassow et al., 2012; Gomes de Melo Coelho et al., 2013a, 2014; Pereira et al., 2013; Schmolesky et al., 2013; Huang et al., 2014; Leckie et al., 2014; Mang et al., 2014; Skriver et al., 2014; Saucedo Marquez et al., 2015; Szuhany et al., 2015; Dinoff et al., 2017; Helm et al., 2017; Mackay et al., 2017; Marinus et al., 2019; de Azevedo et al., 2020; Nicolini et al., 2020). Specifically, acute endurance exercise protocols encompassing graded maximal, moderate-to-high-intensity continuous (~60–80% of age-predicted maximal heart rate or heart rate reserve; ~55–75% of maximal power output as measured with a maximal oxygen uptake test), and high-intensity intermittent (90% of maximal power output) exercise result in a transient increase in blood BDNF in both healthy and clinical populations (Gold et al., 2003; Ferris et al., 2007; Winter et al., 2007; Goekint et al., 2008; Tang et al., 2008; Gustafsson et al., 2009; Laske et al., 2010; Bos et al., 2011; Cho et al., 2012; Heyman et al., 2012; Schmolesky et al., 2013; Mang et al., 2014; Skriver et al., 2014; Saucedo Marquez et al., 2015; Nicolini et al., 2020). Contrary to acute endurance exercise protocols, endurance exercise training programs have yielded inconsistent results. Indeed, while most studies have found that exercise training does not result in permanently elevated basal, peripheral BDNF levels (Schulz et al., 2004; Schiffer et al., 2009; Baker et al., 2010; Erickson et al., 2011; Ruscheweyh et al., 2011; Voss et al., 2013a; Maass et al., 2016; Goldfield et al., 2018, 2019; Gourgouvelis et al., 2018; Nicolini et al., 2019), Zoladz et al. (2008) and Jeon and Ha (2017) determined that a five-week, moderate- and twelve-week, moderate-to-high-intensity, endurance training increased BDNF in physically active, male subjects and adolescent males, respectively. Zoladz et al. (2008) also showed that athletes had higher basal BDNF concentrations than untrained individuals. Leckie et al. (2014) reported that moderate-intensity walking over a year lead to enhanced BDNF levels, but only in individuals older than 65 years of age. Further, Heisz et al. (2017), albeit finding no group differences in BDNF levels between healthy, low-active subjects who underwent training and those who did not, observed that high responders to exercise (i.e., individuals with greater cardiorespiratory fitness gains) had larger BDNF increases following 6 weeks of high-intensity interval training. Lastly, Seifert et al. (2010) found that 3 months of endurance training increased basal, internal jugular venous BDNF in sedentary, healthy males, pointing to an elevated release of BDNF from the brain following exercise training. Based on these findings, it appears that (1) intense and prolonged training (i.e., athletes) lastingly increases basal, peripheral BDNF (Zoladz et al., 2008); that (2) moderate-intensity training might be sufficient to increase basal, peripheral BDNF in physically active (Zoladz et al., 2008) and older (Leckie et al., 2014) individuals but not in low-active individuals (Gourgouvelis et al., 2018); that (3) to enhance basal, peripheral BDNF levels in low-active subjects, the duration of training should be longer than 6 weeks (Heisz et al., 2017; Nicolini et al., 2019); that (4) even though exercise training might not result in an increase in basal, peripheral BDNF, it facilitates increases in blood BDNF after an acute exercise bout (i.e., BDNF increase following a single exercise session is greater after a period of training compared to BDNF increase after a single exercise session prior to training) (Zoladz et al., 2008; Bansi et al., 2013; Szuhany et al., 2015); that (5) training augments the release of BDNF from the brain, although this increase might not be sufficiently large to be detected in peripheral, venous blood collected from the arm (e.g., cubital vein) (Schiffer et al., 2009; Erickson et al., 2011; Ruscheweyh et al., 2011; Voss et al., 2013b; Maass et al., 2016; Heisz et al., 2017; Gourgouvelis et al., 2018; Nicolini et al., 2019). Of note, in healthy individuals, exercise intensity influences the magnitude of BDNF increase, with high-intensity exercise being more effective than low-to-moderate-intensity exercise in elevating BDNF levels (Ferris et al., 2007; Winter et al., 2007; Schmidt-Kassow et al., 2012; Schmolesky et al., 2013; Saucedo Marquez et al., 2015; Enette et al., 2017; Jeon and Ha, 2017; Antunes et al., 2020). Conversely, in clinical populations, even low-to-moderate intensity exercise enhances blood BDNF (Gold et al., 2003; Gustafsson et al., 2009; Laske et al., 2010). Findings from studies investigating whether resistance exercise elevates peripheral BDNF, acutely (i.e., after a single session) or lastingly (i.e., following a training program), are mixed. Goekint et al. (2010) and Correia et al. (2010) found that acute resistance exercise was not effective in elevating peripheral BDNF in healthy subjects, while Yarrow et al. (2010) reported a significant increase in peripheral BDNF following a single session of resistance exercise in a similar cohort. More recently, Marston et al. (2017) also observed that acute resistance exercise transiently elevated blood BDNF levels in healthy subjects. Notably, these authors, however, found that the increase in BDNF was significant only in the resistance-exercise-to-fatigue (i.e., hypertrophy) group involving three sets of ten repetitions with a 60-s recovery between each set (Marston et al., 2017), suggesting that similar to acute endurance exercise, the effect of acute resistance exercise is intensity dependent. Lastly, based on the evidence garnered thus far, resistance training appears to be ineffective in augmenting basal, peripheral BDNF. Specifically, Schiffer et al. (2009) and Goekint et al. (2010) reported no changes in basal, peripheral BDNF levels following resistance training (12 *vs*. 10 weeks) in healthy individuals. Further, Levinger et al. (2008) and Goldfield et al. (2018, 2019) observed similar results in middle-aged subjects with high or low metabolic risk factors and obese and overweight adolescents, respectively. Nonetheless, despite being unable to elevate basal, peripheral BDNF concentrations, resistance training similar to endurance training (Zoladz et al., 2008; Bansi et al., 2013; Szuhany et al., 2015) leads to a robust increase in peripheral BDNF following an acute bout of resistance exercise (i.e., primes BDNF response to acute resistance exercise) (Yarrow et al., 2010).

BDNF is a well-established key regulator of synaptic plasticity (Bramham and Messaoudi, 2005; Kleim et al., 2006; Kuipers and Bramham, 2006; Bekinschtein et al., 2008; Waterhouse and Xu, 2009; Fritsch et al., 2010; Yoshii and Constantine-Paton, 2010; Lu et al., 2014; Gibon and Barker, 2017; Leal et al., 2017; Kowiański et al., 2018), a neural substrate of cognitive function and motor behavior (Rioult-Pedotti et al., 2000; Muellbacher et al., 2002; Doyon and Benali, 2005; Monfils et al., 2005; McConnell et al., 2009; Dayan and Cohen, 2011; Cantarero et al., 2013). As such, it is likely that exercise-induced upregulation of BDNF contributes to enhanced plasticity within the motor system (Gómez-Pinilla et al., 2002), which, in turn, facilitates motor learning and translates into motor improvements such as increased fine motor control (Quaney et al., 2009) (Figure 2). In mice, long-term exercise increases BDNF levels in brain motor areas, such as the primary motor cortex and cerebellum, and improves motor coordination (Inoue et al., 2018). However, in humans, it remains to be determined whether increases in peripheral BDNF following exercise are mechanistically linked to exercise-induced increases in motor plasticity and ultimately to exercise-induced motor gains (e.g., improved mobility, gait, balance, and fine motor control). To date, few studies, all in healthy individuals, have investigated whether elevated blood BDNF is associated with improved motor learning following acute exercise (Mang et al., 2014; Skriver et al., 2014; Helm et al., 2017; Baird et al., 2018). Only Skriver et al. (2014) found a positive correlation. Of note, Baird et al. (2018) did not observe a significant rise in plasma BDNF concentrations following exercise. Lastly, an association between increased serum BDNF and increased motor plasticity (i.e., enhanced TMS-probed corticospinal excitability) after a single bout of exercise has yet to be found (Mang et al., 2014; Nicolini et al., 2020). To determine BDNF contribution to exercise-induced motor improvements, it is thus important that future studies investigate further whether a correlation between exercise-induced changes in BDNF and exercise-induced changes in motor outcomes, encompassing mobility, gait, balance, and motor control, exists following both acute exercise and training in healthy and clinical populations. In addition, it should be established whether, after exercise, there is an association between increases in peripheral BDNF, increases in motor plasticity (e.g., enhanced TMS-probed corticospinal excitability and motor learning), and motor improvements. Findings from these studies are critical to expand our understanding of blood BDNF role in mediating exercise motor benefits via neural mechanisms (i.e., enhanced synaptic plasticity within the motor system).

### Peripheral Brain-Derived Neurotrophic Factor: Caveats and Limitations

**Figure 3:**
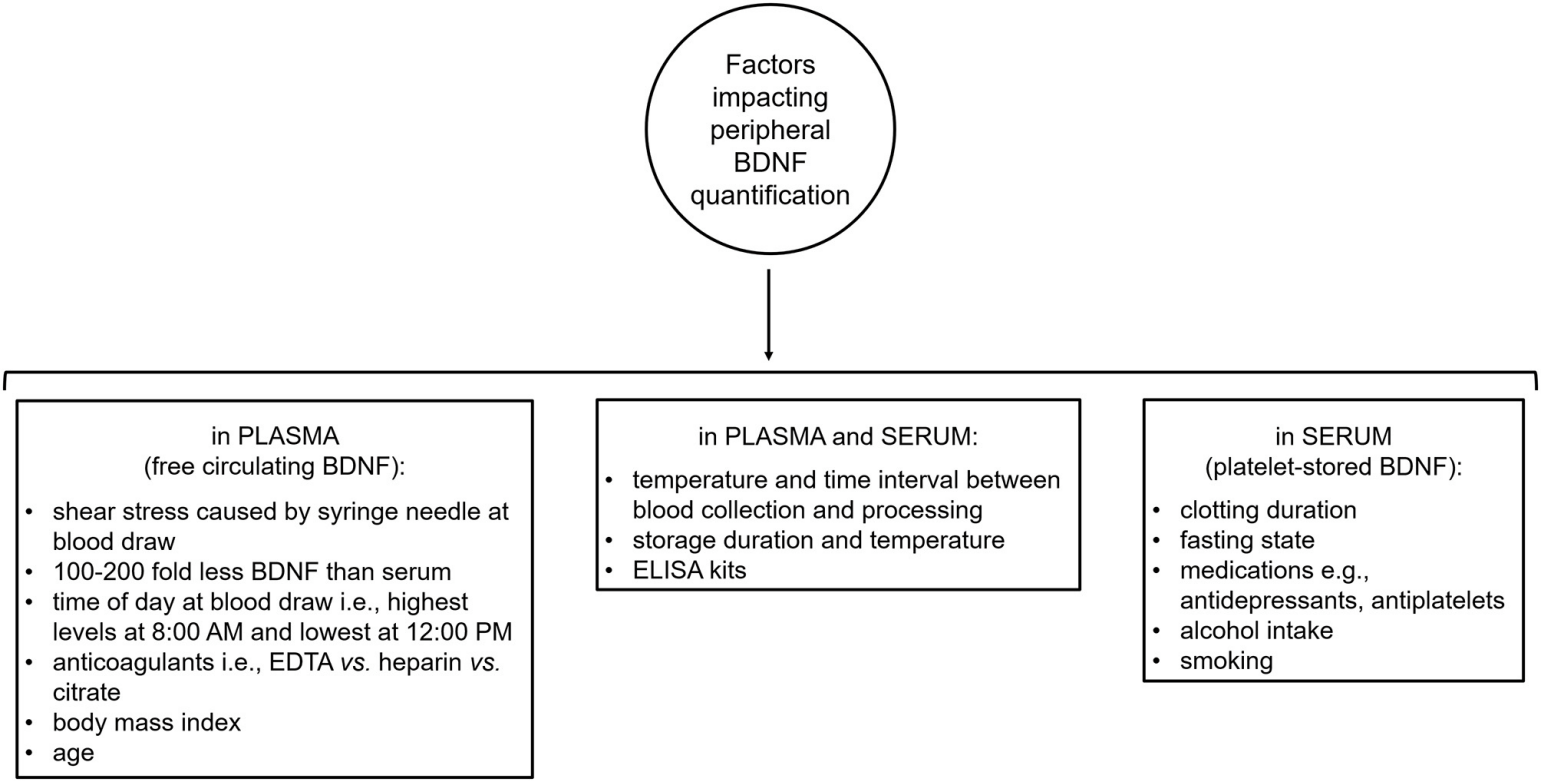
Methodological and non-methodological factors introducing intra- and inter-variability in peripheral brain-derived neurotrophic factor quantification. BDNF, brain-derived neurotrophic factor; EDTA, ethylenediaminetetraacetic acid; ELISA, enzyme-linked immunosorbent assay.

BDNF is present in most human tissues including brain and blood (Pruunsild et al., 2007; Serra-Millàs, 2016). The majority of blood BDNF is stored in platelet granules, from which it is released (degranulation) upon platelet activation (Yamamoto and Gurney, 1990; Fujimura et al., 2002). Non-neural sources of platelet-stored BDNF include vascular human endothelial cells, activated T and B cells and monocytes (Donovan et al., 1995, 2000; Kerschensteiner et al., 1999; Leventhal et al., 1999; Nakahashi et al., 2000). Also, more recently, Chacón-Fernández et al. (2016) found that platelet progenitors, megakaryocytes, express BDNF mRNA transcripts in a pattern similar to neurons. BDNF release from platelets takes place during clotting as supported by reports of a strong correlation between serum BDNF and serum serotonin, which is an indicator of platelet activation (Radka et al., 1996; Fujimura et al., 2002). Notably, shear stress, such as that caused by the syringe needle during blood collection, also induces BDNF release from platelet granules, particularly platelet release of BDNF due to shear stress is proportional to the strength of the stress (Fujimura et al., 2002). Peripheral BDNF can be measured in whole blood, plasma, and serum, however, its levels are between 100 and 200 fold higher in serum than plasma (Rosenfeld et al., 1995; Radka et al., 1996; Fujimura et al., 2002; Gejl et al., 2019). Given the difficulty of assessing brain BDNF concentrations in living humans, the presence of BDNF in blood has attracted considerable interest. Yet, quantification of peripheral BDNF levels is complicated by a number of methodological and non-methodological factors which introduce intra- and inter-individual variability in blood BDNF measures, impacting their reliability and reproducibility across studies (Figure 3). In particular, methodological and non-methodological factors such as different anti-coagulants (e.g., ethylenediaminetetraacetic acid (EDTA) *vs*. heparin *vs*. citrate), temperature and length of time interval between collection of blood samples and their centrifugation, storage duration and temperature, body mass index, and age strongly affect plasma BDNF levels (Lommatzsch et al., 2005; Bus et al., 2011; Tsuchimine et al., 2014; Polyakova et al., 2017). Diurnal changes in plasma BDNF have also been reported with highest levels in the morning (i.e., 8:00 A.M.) and lowest at night (i.e., 12:00 A.M.) (Begliuomini et al., 2008; Piccinni et al., 2008; Pluchino et al., 2009). Similarly, serum BDNF levels are influenced by temperature during the time interval between collection of blood samples and their centrifugation as well as storage duration and temperature (Trajkovska et al., 2007; Bus et al., 2011; Elfving et al., 2012; Amadio et al., 2017). Another important determinant of serum BDNF levels is clotting duration as supported by the evidence that longer clotting duration is associated with higher serum BDNF levels with the lowest concentration being measured after 10 min of clotting and a plateau being reached at ~1 h (Maffioletti et al., 2014; Gejl et al., 2019). Non-technical factors that affect serum BDNF levels include non-fasting state at blood collection, smoking, alcohol intake, medications, such as antidepressant venlafaxine and the antiplatelet medication clopidogrel (Aydemir et al., 2005; Sen et al., 2008; Bus et al., 2011; Stoll et al., 2011). Lastly, ELISA kits contribute to the intra- and inter-individual variability seen in peripheral BDNF concentrations. Indeed, Polacchini et al. (2015) showed that among five, commercially available, sandwich ELISA kits (i.e., Aviscera-Bioscience, Biosensis, Millipore-ChemiKine™, Promega-Emax®, and R&D System-Quantikine®) for BDNF quantification, only one (Biosensis) had minimal inter-assay variability, thereby drawing attention to the need of using the same ELISA kit to measure BDNF across studies to limit result discrepancies.

Serum and plasma BDNF concentrations appear to reflect different pools of BDNF. BDNF in serum comes from platelet storage granules from which it is released during clotting (Fujimura et al., 2002; Maffioletti et al., 2014; Gejl et al., 2019). BDNF in plasma is thought to represent the small amount of free circulating BDNF, as plasma contains few platelets (Radka et al., 1996). During blood collection, however, platelets can release BDNF due to shear stress caused by the syringe needle and increase BDNF levels in plasma (Fujimura et al., 2002). Also, an increase in plasma BDNF can be due to release of BDNF from platelets occurring during the time interval between collection and centrifugation of blood samples (Elfving et al., 2010; Tsuchimine et al., 2014), as some degree of platelet activation and degranulation has been observed even in the presence of anticoagulants (e.g., EDTA-coated tubes) (Engstad et al., 1997; Ahnadi et al., 2003). These methodology-related sources of platelet-derived BDNF contribute to the considerable intra- and inter-individual variability of plasma BDNF measures (~100–7,000 pg/ml), impacting their reliability and reproducibility (Bocchio-Chiavetto et al., 2010; Polacchini et al., 2015; Gejl et al., 2019). Indeed, BDNF quantification in plasma appears to be heavily affected by methodological factors (e.g., shear stress at blood draw, temperature and length of time interval between collection of blood samples and their centrifugation, anticoagulants, storage temperature, and duration), to be greatly dependent on the experimenter, and thus, to ultimately be less reliable than serum BDNF measurements (Elfving et al., 2010; Tsuchimine et al., 2014; Polacchini et al., 2015; Polyakova et al., 2017). Stability of BDNF serum levels over a year and reliability of their measurements have been recently confirmed by Naegelin et al. (2018), who, however, emphasized the need, given individual variations, to examine large cohorts. Taken together, evidence from these studies encourages the use of serum over plasma when measuring peripheral BDNF to evaluate its contribution to exercise-induced motor benefits.

As direct measurements of brain BDNF levels cannot easily be performed in humans, quantification of peripheral BDNF concentrations has been used as a proxy. Animal studies have shown that BDNF crosses the blood-brain barrier (Poduslo and Curran, 1996; Pan et al., 1998; Alcalá-Barraza et al., 2010) and that there is a positive correlation between blood and brain BDNF levels, suggesting that changes in peripheral BDNF might reflect changes in brain BDNF (Karege et al., 2002; Sartorius et al., 2009; Klein et al., 2011). Nevertheless, it currently remains mostly speculative whether, in humans, changes in peripheral BDNF, e.g., following acute exercise, are a reliable proxy of changes in central (brain) BDNF. Measurement of the arterial-to-internal jugular venous concentration difference (*a-v* difference), as an indicator of cerebral outflow, has been used to test whether release of BDNF from the brain contributes to blood BDNF levels. Promisingly, using this approach, three studies have provided evidence for BDNF being released from the brain into the circulation both at rest (Dawood et al., 2007; Krabbe et al., 2007; Rasmussen et al., 2009) and during exercise (Rasmussen et al., 2009). In particular, Krabbe et al. (2007) showed cerebral BDNF output into circulation in healthy men at rest defined as the mean area under the curve for the *a-v* difference being different from zero. Similarly, Dawood et al. (2007) reported a positive internal jugular veno-arterial BDNF plasma concentration gradient as well as higher BDNF levels in plasma obtained from internal jugular venous blood than in plasma obtained from brachial arterial blood, indicating that efflux of BDNF from the brain contributes to peripheral BDNF concentrations at rest in patients with major depressive disorder. Lastly, Rasmussen et al. (2009) confirmed cerebral output of BDNF into circulation via the internal jugular vein at rest in healthy men as indicated by the *a-v* difference being different from zero and showed a two- to three-fold increase in the *a-v* difference (i.e., increased central BDNF outflow) with prolonged exercise (i.e., 4 h of ergometer rowing). Collectively, these findings support the hypothesis that blood BDNF levels reflect brain BDNF levels and the use of peripheral BDNF measures to expand our understanding of the neural mechanisms behind exercise benefits.

## Future Avenues

Although exercise upregulates peripheral BDNF (Knaepen et al., 2010; Zoladz and Pilc, 2010; Gomes de Melo Coelho et al., 2013a; Huang et al., 2014; Szuhany et al., 2015; Dinoff et al., 2017; Mackay et al., 2017; Marinus et al., 2019; de Azevedo et al., 2020), it is still unclear whether increases in peripheral BDNF mediate exercise effects on mobility, gait, balance, and motor control. To date, findings are few and conflicting (Mang et al., 2014; Skriver et al., 2014; Helm et al., 2017). Evaluation of exercise-induced increases in peripheral BDNF, motor plasticity (e.g., enhanced TMS-probed corticospinal excitability and motor learning), and motor outcomes (i.e., improved mobility, gait, balance, and motor control) within a single study is key to advancing our understanding. It is indeed essential to garner evidence at different levels of analysis (i.e., systemic, molecular, behavioral) within the same study to unveil the mechanistic link between exercise and motor improvements, and to thus, gain the knowledge needed to successfully employ exercise protocols in preventing, delaying, and off-setting age- and disease-related motor deficits.

A single nucleotide polymorphism (Val66Met, rs6265) in the BDNF gene, causing a valine-to-methionine substitution, reduces activity-dependent release of BDNF (Egan et al., 2003) and could, thereby, attenuate BNDF-driven, priming effects of exercise on neuroplasticity, motor learning, and, ultimately, on motor outcomes (i.e., mobility, gait, balance, and motor control). Current evidence is limited and mixed. Andrews et al. (2020) showed that BDNF Val66Met polymorphism reduced exercise priming effects on plasticity within the primary motor cortex, while McDonnell et al. (2013) and Singh et al. (2014) reported no effect. However, Singh et al. (2014) might not have been adequately powered (*n* = 6, Met carriers; *n* = 6, Val/Val) to detect whether BDNF genotype impacts exercise effects on motor plasticity, measured using different repetitive transcranial magnetic stimulation paradigms. Of note, Met carriers showed a trend toward a stronger reduction in GABA_A_-mediated inhibition (i.e., lower short-interval intracortical inhibition) than Val/Val homozygotes and no change in GABA_B_-mediated inhibition (i.e., long-interval intracortical inhibition) (Singh et al., 2014), underlining the need to investigate further the effects of BDNF Val66Met polymorphism on exercise priming of motor plasticity. Lastly, although there are two reports that BDNF genotype does not affect exercise effects on motor learning (Helm et al., 2017; Mang et al., 2017), it remains to be assessed whether it attenuates exercise-induced motor improvements in healthy, aging, or clinical populations. Identifying potential determinants of individual variation, such as BDNF Val66Met polymorphism, is important for the design of personalized exercise strategies aimed at maximizing priming of neuroplasticity and thus motor improvements in both physiological and rehabilitative settings.

As methodological and non-methodological factors influence quantification of peripheral BDNF concentrations (Aydemir et al., 2005; Lommatzsch et al., 2005; Trajkovska et al., 2007; Begliuomini et al., 2008; Piccinni et al., 2008; Sen et al., 2008; Pluchino et al., 2009; Bus et al., 2011; Choi et al., 2011; Stoll et al., 2011; Maffioletti et al., 2014; Tsuchimine et al., 2014; Polacchini et al., 2015; Amadio et al., 2017; Polyakova et al., 2017; Naegelin et al., 2018; Gejl et al., 2019), the lack of a standardized protocol currently hinders result interpretation and comparison and leads to discrepancies among studies. To limit variability in blood BDNF measures and reduce inconsistencies, future studies should aim at developing a standardized, reliable protocol for peripheral BDNF quantification. By increasing result reproducibility, such a protocol would help draw reliable conclusions on whether peripheral BDNF mediates exercise-related motor benefits.

Exercise might not only increase total levels of BDNF, but also the speed of BDNF release. In other words, BDNF might be released faster into serum during clotting after exercise than following a period of rest of comparable length. Interestingly, Gejl et al. (2019), despite failing to find a significant correlation between cardiorespiratory fitness and serum BDNF levels, observed a switch from a positive correlation at 30 min of clotting to a negative one at 60 min and at longer clotting times (180, 240, and 300 min) as well as a negative correlation between cardiorespiratory fitness and the difference in serum BDNF measured at 30 and at 60 min of clotting. These findings suggest that greater cardiorespiratory fitness is associated with a faster initial release of BDNF into serum during clotting and with less BDNF being released at 60 min and at longer clotting times (Gejl et al., 2019). Effects of exercise (i.e., acute and training) on the rate of BDNF should be further assessed in future studies.

## Discussion

Being a key mediator of neuroplasticity (Lu et al., 2014; Gibon and Barker, 2017; Leal et al., 2017; Kowiański et al., 2018), which has been shown to underpin motor learning (Rioult-Pedotti et al., 2000; Muellbacher et al., 2002; Doyon and Benali, 2005; Monfils et al., 2005; McConnell et al., 2009; Dayan and Cohen, 2011; Cantarero et al., 2013), BDNF is likely to play an important role in mediating the beneficial effects of exercise on mobility, gait, balance, and motor control. Currently, however, only two studies have investigated whether BDNF contributes to exercise-induced motor improvements (Mang et al., 2014; Skriver et al., 2014). Skriver et al. (2014) reported a relationship between BDNF increases and gains in motor skill acquisition and retention following exercise, while Mang et al. (2014) failed to find one. It is thereby clear that it needs to be further investigated whether BDNF is mechanistically linked to exercise-induced motor benefits. To this end, given the difficulties of obtaining direct measures of brain BDNF concentrations in humans, it is crucial to be able to reliably measure peripheral BDNF levels so that results are reproducible and can be compared among studies. Intra- and inter-individual variability in peripheral BDNF concentrations (Bocchio-Chiavetto et al., 2010; Fernandes et al., 2011; Suliman et al., 2013) currently hinders interpretation of findings, result comparisons, and the ability to draw reliable conclusions, impeding our understanding of how exercise promotes neuroplasticity and thus improves mobility, gait, balance, and motor control. Methodological and non-methodological (e.g., sociodemographic, lifestyle) factors that affect quantification of BDNF blood levels and are a source of discrepancies among studies include temperature and length of time between collection of blood samples and their centrifugation, centrifugation speed and duration, storage temperature and duration, number of freeze/thaw cycles, ELISA kits used for quantification, non-fasting state at blood draw, time of day at which blood samples are collected, medications, age, body mass index, menstrual cycle phase, smoking, and alcohol intake (Aydemir et al., 2005; Lommatzsch et al., 2005; Trajkovska et al., 2007; Begliuomini et al., 2008; Piccinni et al., 2008; Sen et al., 2008; Pluchino et al., 2009; Elfving et al., 2010, 2012; Bus et al., 2011; Choi et al., 2011; Stoll et al., 2011; Maffioletti et al., 2014; Tsuchimine et al., 2014; Polacchini et al., 2015; Amadio et al., 2017; Polyakova et al., 2017; Naegelin et al., 2018; Gejl et al., 2019). The evidence that methodological factors profoundly influence quantification of peripheral BDNF calls attention to the importance of exercising caution in the methodology used to measure blood BDNF concentrations and to the pressing need for a standardized protocol across studies. Indeed, a standardized protocol encompassing all steps of peripheral BDNF detection, including participant selection criteria (e.g., body mass index, age, medications, smoking, alcohol intake), blood collection (e.g., time of day, fasting state, time interval between collection and processing, temperature during this interval), processing (e.g., clotting duration, centrifugation speed, and duration), storage (e.g., temperature, duration), and BDNF quantification (i.e., ELISA kit used), should be developed and used for quantification of peripheral BDNF concentrations across studies. Based on findings garnered thus far, it appears that to reduce intra- and inter-variability in peripheral BDNF measures serum should be preferred over plasma (Elfving et al., 2010; Polacchini et al., 2015; Polyakova et al., 2017; Naegelin et al., 2018), serum samples should be allowed to clot a minimum of 30 min up to 1 h (Maffioletti et al., 2014; Gejl et al., 2019), Biosensis ELISA kit should be used for BDNF quantification (Polacchini et al., 2015), and blood should be collected in the morning (i.e., 8:00 A.M.) (Begliuomini et al., 2008; Piccinni et al., 2008; Pluchino et al., 2009; Choi et al., 2011) from fasting participants (Table 1; Bus et al., 2011). In addition, medications, body mass index, age, menstrual cycle phase, smoking, and alcohol intake should be taken into account when recruiting participants (Aydemir et al., 2005; Lommatzsch et al., 2005; El-Gharbawy et al., 2006; Ziegenhorn et al., 2007; Sen et al., 2008; Pluchino et al., 2009; Bus et al., 2011; Stoll et al., 2011; Cho et al., 2012; Elfving et al., 2012; Jamal et al., 2015). By reducing the considerable result variability in blood BDNF concentrations and thus providing reproducible results, such a protocol is highly valuable in expanding our understanding of how exercise improves mobility, gait, balance, and motor control in physiological and rehabilitative settings.

**Table 1:** Recommendations based on current evidence to reduce intra- and inter-variability in peripheral brain-derived neurotrophic factor quantification.

**Best practice**	**Reference**
Serum over plasma	Elfving et al., 2010
	Polacchini et al., 2015
	Polyakova et al., 2017
	Naegelin et al., 2018
Clotting time from 30 min to 1 h	Maffioletti et al., 2014
	Gejl et al., 2019
Biosensis ELISA kit	Polacchini et al., 2015
Blood collection time between 7:00 A.M. and 8:00 A.M.	Begliuomini et al., 2008
	Piccinni et al., 2008
	Pluchino et al., 2009
	Choi et al., 2011
12-h fast prior to blood collection	Bus et al., 2011

## Author Contributions

CN wrote, reviewed, and edited the manuscript. AJN funded, reviewed, and edited the manuscript. All authors have read and approved the final manuscript.

## Conflict of Interest

The authors declare that the research was conducted in the absence of any commercial or financial relationships that could be construed as a potential conflict of interest.
